# Supplementary material for: University students’ perspectives, planned uptake, and hesitancy regarding the COVID-19 vaccine: A multi-methods study
Source: PLoS One. 2021 Aug 3;16(8):e0255447. doi: 10.1371/journal.pone.0255447 (PMC8330905; doi:10.1371/journal.pone.0255447)
Supplement: S1 File — June/July 2020 and September/October 2020 survey questions, semi-structured interview questions. (DOCX) [file pone.0255447.s001.docx]

**Survey Guide: June/July 2020**

1. What is your age? (write in box)
2. To which gender identity do you most identify?
   1. Male
   2. Female
   3. Gender variant/non-conforming
   4. Prefer not to answer
   5. Prefer to self describe (write in box below)
3. How do you describe your ethnicity? (box provided to write in answer)
4. What is your year of study at the University of Toronto?
   1. Undergraduate year 1
   2. Undergraduate year 2
   3. Undergraduate year 3
   4. Undergraduate year 4
   5. Undergraduate year 5
   6. Undergraduate year other
   7. Graduate
5. What is your program of study at the University of Toronto? (write in box)
6. What is the location of your permanent residence?
   1. Greater Toronto Area (GTA)
   2. Southwestern Ontario (other than the GTA)
   3. Northern Ontario
   4. Quebec
   5. Eastern Canadian provinces
   6. Western Canadian provinces
   7. Eastern Ontario
   8. Other (write in below)
7. What is your household income (if you support yourself) or your family’s annual household income, if they provide more than 50% of your income support (CAD)?
   1. Less than $24 999
   2. $25 000 to $49 999
   3. $50 000 to $74 999
   4. $75 000 to $99 999
   5. $100 000 to $124 999
   6. $125 000 to $149 999
   7. Greater than $150 000
8. Have you been personally affected by the current COVID-19 outbreak through the illness of yourself or an immediate family member?
   1. Yes
   2. No
9. Do you have regular access to social media (e.g., Instagram, Facebook, Twitter, TikTok) as a news source?
   1. Yes (if yes, move to Questions 10-12)
   2. No (if no, move to Question 13)
10. In a typical week, how much time per DAY (in minutes) do you spend using social media for general use? (write in box)
11. In a typical week, how much time per DAY (in minutes) do you spend using social media as a source for COVID-19 health-related information?
12. How successful do you feel social media is in bringing clear, concise, and unbiased information about the COVID-19 outbreak?
    1. 7-point Likert scale (1: unsuccessful, 7: successful)
13. Do you have regular access to internet news sources (e.g., CBC, BBC, CNN, Globe and Mail) as a news source?)
    1. Yes (if yes, move to Questions 14-16)
    2. No (if no, move to Question 17)
14. In a typical week, how much time per DAY (in minutes) do you spend using internet news sources for general use? (write in box)
15. In a typical week, how much time per DAY (in minutes) do you spend using internet news sources as a source for COVID-19 health-related information? (write in box)
16. How successful do you feel internet news sources are in bringing clear, concise, and unbiased information about the COVID-19 outbreak?
    1. 7-point Likert scale (1: unsuccessful, 7: successful)
17. Do you have regular access to the radio as a news source?
    1. Yes (if yes, move to Questions 18-20)
    2. No (if no, move to Question 21)
18. In a typical week, how much time per DAY (in minutes) do you spend using the radio for general use? (write in box)
19. In a typical week, how much time per DAY (in minutes) do you spend using the radio as a source for COVID-19 health-related information? (write in box)
20. How successful do you feel the radio is in bringing clear, concise, and unbiased information about the COVID-19 outbreak?
    1. 7-point Likert scale (1: unsuccessful, 7: successful)
21. Do you have regular access to television as a news source?
    1. Yes (if yes, move to Questions 22-24)
    2. No (if no, move to Question 25)
22. In a typical week, how much time per DAY (in minutes) do you spend using television for general use? (write in box)
23. In a typical week, how much time per DAY (in minutes) do you spend using television as a source for COVID-19 health-related information? (write in box)
24. How successful do you feel television is in bringing clear, concise, and unbiased information about the COVID-19 outbreak?
    1. 7-point Likert scale (1: unsuccessful, 7: successful)
25. Do you have regular access to magazines as a news source?
    1. Yes (if yes, move to Questions 26-28)
    2. No (if no, move to Question 29)
26. In a typical week, how much time per DAY (in minutes) do you spend using magazines for general use? (write in box)
27. In a typical week, how much time per DAY (in minutes) do you spend using magazines as a source for COVID-19 health-related information? (write in box)
28. How successful do you feel magazines are in bringing clear, concise, and unbiased information about the COVID-19 outbreak?
    1. 7-point Likert scale (1: unsuccessful, 7: successful)
29. How anxious or fearful of acquiring COVID-19 are you after hearing or reading a news report updating the outbreak?
    1. 7-point Likert scale (1: not fearful/anxious, 7: very fearful/anxious)
30. How much time does the media spend covering the COVID-19 outbreak?
    1. 7-point Likert scale (1: not enough, 7: too much)
31. How severe a threat is COVID-19?
    1. 7-point Likert scale (1: not severe, 7: very severe)
32. Do you think you are at risk of contracting COVID-19?
    1. Yes
    2. No
33. Why do you think you are or are not at risk of contracting COVID-19? (write in box)
34. Which form of media made you feel the most anxiety and fear about becoming infected with COVID-19?
    1. Social media
    2. Internet news source
    3. Radio
    4. Television
    5. Magazines
    6. Other (write in below)
35. Has the news coverage of COVID-19 influenced your day-to-day behaviour? (Choose all that apply)
    1. No change
    2. Cancelled or changed travel plans
    3. I wear a mask some or all of the time
    4. I am washing my hands more
    5. I am using hand sanitizer
    6. I am social distancing
    7. I am self isolating
    8. I am cleaning more
    9. I have bought extra food/supplies
    10. I have gone to or plan to go to the doctor for help/advice
    11. I have gone to or plan to go to the hospital for help/advice
    12. I have called public health
    13. I have been tested for COVID-19
    14. Other (write in box below)
36. What do you think are the main factors influencing the spread of COVID-19? (write in box)
37. What effects have the government-mandated social distancing and non-essential closures had on your life (employment, financial, social, etc.)? (write in box)
38. How are the social distancing rules and the pandemic in general affecting your mental health? (write in box)
39. What strategies, support systems, or programs for mental health or otherwise (economic, social) would you find beneficial at this time (what could the local, provincial, or federal government be doing to help you)? (write in box)
40. What is your opinion of the current plan to re-open your province/city/town? What do you feel is being done well and what could be done differently? (write in box)
41. Have you experienced, heard of, or witnessed any racism towards Asian Canadians during the pandemic? If so, what have you seen/heard/read? (write in box)
42. If a vaccine for COVID-19 were to become available, would you want to get it?
    1. Yes
    2. No
    3. Expand on your answer (write in box)
43. If a vaccine trial was run in Canada, would you volunteer to be part of the trial?
    1. Yes
    2. No
    3. Expand on your answer (write in box)
44. Is there anything else you would like to say about the COVID-19 outbreak or your response that we did not ask? (write in box)
45. Did you participate in the first round of this survey (March/April 2020)?
    1. Yes
    2. No
46. As part of our research we are looking for participants to be interviewed about their perceptions of COVID-19 over videoconferencing. All interviews will be confidential. If you are interested in receiving more information about the interview, please write your email into the box below and we will contact you. Providing your email does NOT mean you must participate.
47. Thank you for participating in our survey! If you would like to be entered into a draw for one of three $50 gift cards please enter your email below. Please press “Submit” below to complete the survey.

**Survey Guide: September/October 2020**

1. What is your age? (write in box)
2. To which gender identity do you most identify?
   1. Male
   2. Female
   3. Gender variant/non-conforming
   4. Prefer not to answer
   5. Prefer to self describe (write in box below)
3. How do you describe your ethnicity? (write in box)
4. What is your year of study at the University of Toronto?
   1. Undergraduate year 1
   2. Undergraduate year 2
   3. Undergraduate year 3
   4. Undergraduate year 4
   5. Undergraduate year 5
   6. Undergraduate year other
   7. Graduate
5. What is your program of study at the University of Toronto? (write in box)
6. What is the location of your permanent residence?
   1. Greater Toronto Area (GTA)
   2. Southwestern Ontario (other than the GTA)
   3. Northern Ontario
   4. Quebec
   5. Eastern Canadian provinces
   6. Western Canadian provinces
   7. Eastern Ontario
   8. Other (write in below)
7. What is your household income (if you support yourself) or your family’s annual household income, if they provide more than 50% of your income support (CAD)?
   1. Less than $24 999
   2. $25 000 to $49 999
   3. $50 000 to $74 999
   4. $75 000 to $99 999
   5. $100 000 to $124 999
   6. $125 000 to $149 999
   7. Greater than $150 000
8. Have you been personally affected by the current COVID-19 outbreak through the illness of yourself or an immediate family member?
   1. Yes
   2. No
9. Do you have regular access to social media (e.g., Instagram, Facebook, Twitter, TikTok) as a news source?
   1. Yes (if yes, move to Questions 10-12)
   2. No (if no, move to Question 13)
10. In a typical week, how much time per DAY (in minutes) do you spend using social media for general use? (write in box)
11. In a typical week, how much time per DAY (in minutes) do you spend using social media as a source for COVID-19 health-related information?
12. How successful do you feel social media is in bringing clear, concise, and unbiased information about the COVID-19 outbreak?
    1. 7-point Likert scale (1: unsuccessful, 7: successful)
13. Do you have regular access to internet news sources (e.g., CBC, BBC, CNN, Globe and Mail) as a news source?)
    1. Yes (if yes, move to Questions 14-16)
    2. No (if no, move to Question 17)
14. In a typical week, how much time per DAY (in minutes) do you spend using internet news sources for general use? (write in box)
15. In a typical week, how much time per DAY (in minutes) do you spend using internet news sources as a source for COVID-19 health-related information? (write in box)
16. How successful do you feel internet news sources are in bringing clear, concise, and unbiased information about the COVID-19 outbreak?
    1. 7-point Likert scale (1: unsuccessful, 7: successful)
17. Do you have regular access to the radio as a news source?
    1. Yes (if yes, move to Questions 18-20)
    2. No (if no, move to Question 21)
18. In a typical week, how much time per DAY (in minutes) do you spend using the radio for general use? (write in box)
19. In a typical week, how much time per DAY (in minutes) do you spend using the radio as a source for COVID-19 health-related information? (write in box)
20. How successful do you feel the radio is in bringing clear, concise, and unbiased information about the COVID-19 outbreak?
    1. 7-point Likert scale (1: unsuccessful, 7: successful)
21. Do you have regular access to television as a news source?
    1. Yes (if yes, move to Questions 22-24)
    2. No (if no, move to Question 25)
22. In a typical week, how much time per DAY (in minutes) do you spend using television for general use? (write in box)
23. In a typical week, how much time per DAY (in minutes) do you spend using television as a source for COVID-19 health-related information? (write in box)
24. How successful do you feel television is in bringing clear, concise, and unbiased information about the COVID-19 outbreak?
    1. 7-point Likert scale (1: unsuccessful, 7: successful)
25. Do you have regular access to magazines as a news source?
    1. Yes (if yes, move to Questions 26-28)
    2. No (if no, move to Question 29)
26. In a typical week, how much time per DAY (in minutes) do you spend using magazines for general use? (write in box)
27. In a typical week, how much time per DAY (in minutes) do you spend using magazines as a source for COVID-19 health-related information? (write in box)
28. How successful do you feel magazines are in bringing clear, concise, and unbiased information about the COVID-19 outbreak?
    1. 7-point Likert scale (1: unsuccessful, 7: successful)
29. How anxious or fearful of acquiring COVID-19 are you after hearing or reading a news report updating the outbreak?
    1. 7-point Likert scale (1: not fearful/anxious, 7: very fearful/anxious)
30. How much time does the media spend covering the COVID-19 outbreak?
    1. 7-point Likert scale (1: not enough, 7: too much)
31. How severe a threat is COVID-19?
    1. 7-point Likert scale (1: not severe, 7: very severe)
32. Do you think you are at risk of contracting COVID-19?
    1. Yes
    2. No
33. Why do you think you are or are not at risk of contracting COVID-19? (write in box)
34. Which form of media made you feel the most anxiety and fear about becoming infected with COVID-19?
    1. Social media
    2. Internet news source
    3. Radio
    4. Television
    5. Magazines
    6. Other (write in below)
35. Has the news coverage of COVID-19 influenced your day-to-day behaviour? (Choose all that apply)
    1. No change
    2. Cancelled or changed travel plans
    3. I wear a mask some or all of the time
    4. I am washing my hands more
    5. I am using hand sanitizer
    6. I am social distancing
    7. I am self isolating
    8. I am cleaning more
    9. I have bought extra food/supplies
    10. I have gone to or plan to go to the doctor for help/advice
    11. I have gone to or plan to go to the hospital for help/advice
    12. I have called public health
    13. I have been tested for COVID-19
    14. I have chosen to get a COVID-19 test
    15. Other (write in box below)
36. What do you think are the main factors influencing the spread of COVID-19? (write in box)
37. What effects have the government-mandated social distancing and non-essential closures had on your life (employment, financial, social, etc.)? (write in box)
38. How are the social distancing rules and the pandemic in general affecting your mental health? (write in box)
39. What strategies, support systems, or programs for mental health or otherwise (economic, social) would you find beneficial at this time (what could the local, provincial, or federal government be doing to help you)? (write in box)
40. What is your opinion of the current plan to re-open your province/city/town? What do you feel is being done well and what could be done differently? (write in box)
41. Have you experienced, heard of, or witnessed any racism towards Asian Canadians during the pandemic? If so, what have you seen/heard/read? (write in box)
42. Do you usually get the seasonal flu vaccine?
    1. Yes (if yes, move to Question 43)
    2. No (if no, move to Question 44)
43. If yes, why? (Select all that apply)
    1. To avoid catching the flu
    2. My doctor recommends that I get it
    3. To avoid illness
    4. It is safe
    5. Worried about becoming seriously ill
    6. I always get the seasonal flu shot
    7. I live with people who are high risk
    8. I am high risk
    9. I am required to because of my job
    10. Other (write in below)
44. If no, why? (Select all that apply)
    1. It will not work
    2. Worried it will cause serious side effects
    3. Worried it will cause bothersome side effects
    4. Worried it will give me the flu
    5. It is not safe
    6. I am not at risk of catching the flu
    7. I don’t know where I would get it
    8. Other (write in below)
45. Did you get the seasonal flu vaccine last year?
    1. Yes
    2. No
    3. Can’t remember
46. Do you plan to get the seasonal flu vaccine this year?
    1. Yes
    2. No
    3. Undecided
47. If a vaccine for COVID-19 were to become available, would you want to get it?
    1. Yes (if yes, move to Question 48)
    2. No (if no, move to Question 49)
48. If yes, why? (Select all that apply)
    1. To avoid catching COVID-19
    2. To avoid illness
    3. It is safe
    4. Worried about becoming seriously ill
    5. COVID-19 is deadlier than the seasonal flu
    6. I always get the seasonal flu shot
    7. I live with people who are high risk
    8. I am high risk
    9. I will be required to because of my job
    10. Other (write in below)
49. If no, why? (Select all that apply)
    1. It will not work
    2. Insufficient testing
    3. Worried it will cause serious side effects
    4. Worried it will cause bothersome side effects
    5. Worried it would give me COVID-19
    6. It is not safe
    7. I am not at risk of catching COVID-19
    8. I don’t know where I would get it
    9. Other (write in below)
50. If a vaccine trial was run in Canada, would you volunteer to be part of the trial?
    1. Yes
    2. No
    3. Expand on your answer (write in box)
51. If your doctor or pharmacist recommended getting the COVID-19 vaccine would that encourage you to get it?
    1. Yes
    2. No
    3. Undecided
52. Did you participate in the first round (March/April 2020) or second round (June/July 2020) of this survey? Select all that apply.
    1. Neither
    2. First round (March/April)
    3. Second round (June/July)
53. As part of our research we are looking for participants to be interviewed about their perceptions of COVID-19 over videoconferencing. All interviews will be confidential. If you are interested in receiving more information about the interview, please write your email into the box below and we will contact you. Providing your email does NOT mean you must participate.
54. Thank you for participating in our survey! If you would like to be entered into a draw for one of three $50 gift cards please enter your email below. Please press “Submit” below to complete the survey.

*Questions concerning media use are adapted from Bergeron and Sanchez (2005). Options regarding reasons for accepting or rejecting vaccines are adapted from Ramsey and Marczinski (2011).*

# INTERVIEW QUESTIONS

1. Where have you gotten information on COVID-19?
   1. Have you gone looking for information? If so where and why? If not, why not?
   2. Are you receiving it passively? Where are you getting it?
   3. If getting it on social media, are you looking it up or just unable to avoid it? How do you feel about the information you are getting on social media?
   4. Do you go looking on news sites, or just follow ones from social media?
   5. Your news/updates: are you getting them locally, from other provinces, Canada, around the world?
   6. Are you spending more/less/the same time seeking information about COVID than you were over the past year?
   7. [if they haven’t already spoken about vaccines] What kind of information have you seen about vaccines? Where are you getting your information about vaccines?
   8. What would you have liked to know that wasn’t available?
2. Are you concerned about COVID-19? Why or why not?
   1. How serious is this virus? What makes you think that? Has your perception of the seriousness changed over this past year?
   2. Do you feel you are susceptible? – explain why or why not
   3. Have you had a COVID-19 test?
   4. Are you concerned about the variants? [if they haven’t brought this up so far]
   5. Have you had a personal experience with COVID-19? Family members? Friends?
   6. When do you think this will end? (Be totally gone? Be less of a problem?)
3. Are you doing anything to avoid contracting COVID-19?
   1. Are you still practicing social distancing? And how is it affecting your life?
   2. Are you in a hotspot? What is your reaction to this (are you surprised that you are/aren’t, why)?
   3. Is social distancing affecting your mental health? If so, how?
      1. Have you accessed any services during the pandemic, such as mental health services, food banks, or signed up for any benefit programs? Or EI?
      2. Did you use any of these before?
   4. Have you found that you’ve increased your use of alcohol or drugs including cannabis during this time?
   5. Are you working right now? How has social distancing affected your work?
   6. Financial (if not brought up under previous questions)
      1. Where does your income come from?
      2. Does social distancing affect your ability to work? (next few weeks/months)
      3. Will this impact you for next year?
4. Do you think the government’s response to COVID-19 is adequate? What, if anything, should be done differently?
   1. If the answer is “more supports for people”: what kind of supports would be beneficial?
5. What do you think of how your university has been handling things lately? How was the past semester for you?
6. Are you aware of any racist incidents toward the Asian-Canadian and/or Chinese communities linked to the COVID-19 pandemic?
7. If/when there is a vaccine available for COVID-19, would you get it? (June/July version)
8. Have you received your COVID-19 vaccine and/or do you plan to receive it when you become eligible? (September/October 2020 version)
   1. If not, what are your specific concerns?
   2. Which of the available vaccines would you be willing to get?
9. What do you miss most about the pre-pandemic period?
10. What are you looking forward to?
11. Is there anything else you wanted to tell us that we haven’t asked you?
