# Supplementary material for: University students’ perspectives, planned uptake, and hesitancy regarding the COVID-19 vaccine: A multi-methods study
Source: PLoS One. 2021 Aug 3;16(8):e0255447. doi: 10.1371/journal.pone.0255447 (PMC8330905; doi:10.1371/journal.pone.0255447)
Supplement: Attachment — Submitted filename: Response to Reviewers.docx [file pone.0255447.s002.docx]

June 25, 2021

**Response to Reviewers**

Thank you to the Reviewers and the Academic Editor for their reviews and comments. We appreciate the time spent assessing this manuscript and hope our revisions have fully addressed the identified points. The response/actions to the comments are noted below in italics. All four authors have approved the following changes to the manuscript.

Please include a copy of the interview guide used in the study, in both the original language and English, as Supporting Information, or include a citation if it has been published previously.

*The June and September survey questions and interview guides are included as Supporting Information.*

**Editor Comments**

1. Results. Please edit down your presentation of the interview data. I would recommend removing table 7 in favor of just a paragraph of general descriptives of the sample. Also, it is not necessary to identify each participant alongside the quotes. i would strongly urge you to consider placing the quotes into a table or text box organized by the themes identified in your text.

*Thank you for these suggestions. All references in parentheses to specific participant numbers have been removed from the text. Table 7 has been removed and replaced with a paragraph summarizing the interview participant sample demographics. All interviewee quotations have been removed from the body text and organized into a table (new Table 7) based upon the themes identified in the Results.*

2. Results. Table 1, please clarify your label "severity." I believe this is referring to respondents' perception of the severity of COVID, but coming right after "anxiety" it gets confusing as a single word label.

*Table 1 has been adjusted so that Anxiety now reads “Self-reported Anxiety” and Severity now reads “Perception of COVID-19’s Severity” to clarify that these are separate sets of results.*

3. Results. line 239. Your description of students not personally affected being 0.369 times less likely is the opposite of how it is presented in the table and the use of a fractional reduced likelihood is awkwardly worded.

*Thank you for catching this. The text has been revised to accurately reflect what is presented in Table 5.*

**Reviewer 1**

1, The presentation of Table 4 and 5 should be revised, as usual appearance in a scientific paper.

*Tables 4 and 5 have been revised to align with APA formatting for reporting logistic regression.*

2, I suggest the qualitative results should be revised and refined.

*Following the comments provided by the Academic Editor, and as detailed above, the references to specific participants have been removed from the main text, Table 7 was removed and replaced with a paragraph summarizing the interview participant sample demographics, and all interviewee quotations have been removed from the text and placed into a new Table 7, organized by the themes identified in the Results.*

**Reviewer 2**

1. The title of this study is "University students' perspectives and hesitancy regarding the COVID-19 vaccine: a multi-methods study," however the elaboration on hesitancy part is insufficient. Further elaboration on the issue in the study, particularly in the discussion and conclusion, is necessary to better fit the title.

*Thank you for this point. We have adjusted the title to “University students’ perspectives,* ***planned uptake****, and hesitancy regarding the COVID-19 vaccine: a multi-methods study” in order to better reflect the aims and content of the paper. The subsection “Qualitative results for vaccine uptake or resistance” has been revised to “Qualitative results for vaccine uptake or hesitancy” and the subsection “Reasons for not receiving the vaccine” was revised to “Reasons for vaccine hesitancy.” The section discussing reasons for vaccine hesitancy has been revised to explicitly state “there were vaccine hesitancy concerns raised as perceived barriers within the HBM.” The speed of development was the key fear expressed by survey participants, which is discussed and placed into the wider context of the limited existing literature.*

2. In regards to the likeliness of university students to get COVID-19 vaccine once it's available, the authors did not include price and regulation as predictors or factors that might influence decision to get vaccinated. In fact, these two factors have been shown to be associated with vaccination uptake in previous investigations. Wether or not the COVID-19 vaccine will be available for free, and if it will be made compulsory by the government might influence the decision of this particular population to get themselves vaccinated. In my opinion, it would be great if the authors could explain why they did not include the two factors in the introduction part (it was explained briefly in the methods section, but no justification of why the two items were not included in the survey).

*This is an important point that is dependent upon the national context in which this research was conducted. Regarding vaccine cost, routine vaccinations for babies, children, and adults are offered free of charge across Canada. Seasonal flu shots are free and the mass immunization program for the H1N1 pandemic in 2009 provided free vaccines for all Canadians who wanted a vaccine. We did not consider cost as a driving factor because the universal health care system in Canada covers the cost of both routine and pandemic vaccines. We also did not consider government mandating of the vaccine because there are no mandated vaccines in Canada (though two provinces have specific rules regarding vaccines for school-aged children to register in public schools, which have been noted in the text. They do, however, have medical and philosophical exemptions.) Survey participants spontaneously offered thoughts regarding making the vaccine mandatory, which has been noted in the results. We added an explanation (in the Methods section, subsection on Survey questions) as to why cost and mandatory vaccines were not considered as potential drivers of the survey participants’ answers. Three new citations have been added to the manuscript as part of this revision (#27-29).*
